# Regulation of UHRF1 acetylation by TIP60 is important for colon cancer cell proliferation

**DOI:** 10.1007/s13258-022-01298-x

**Published:** 2022-08-11

**Authors:** Ye Joo Hong, Junyoung Park, Ja Young Hahm, Song Hyun Kim, Dong Ho Lee, Kwon-Sik Park, Sang-Beom Seo

**Affiliations:** 1 Department of Life Science, College of Natural Sciences, Chung-Ang University, 06974 Seoul, South Korea; 2 Da Vinci College of General Education, Chung-Ang University, 06974 Seoul, South Korea; 3 Department of Microbiology, Immunology, and Cancer Biology, University of Virginia School of Medicine, 22908 Charlottesville, VA USA

**Keywords:** UHRF1, TIP60, Acetylation, Proliferation, Colon cancer

## Abstract

**Background:**

Ubiquitin-like with PHD and RING finger domains 1 (UHRF1) is upregulated in colon cancer cells and associated with silencing tumor suppressor genes (TSGs) to promote colon cancer cell proliferation.

**Objective:**

To investigate epigenetic modification of UHRF1 by TIP60. Whether UHRF1 acetylation by TIP60 can induce cell proliferation in colon cancer cells.

**Methods:**

Acetylation sites of UHRF1 by TIP60 was predicted by ASEB (Acetylation Set Enrichment Based) method and identified by immunoprecipitation assay using anti-pan-acetyl lysine antibody and *in vitro* acetylation assay. Based on this method, UHRF1 acetylation-deficient mimic 4KR (K644R, K646R, K648R, K650R) mutant was generated to investigate effects of UHRF1 acetylation by TIP60. shRNA system was used to generate stable knockdown cell line of UHRF1. With transient transfection of UHRF1 WT and 4KR, the effects of UHRF1 4KR mutant on Jun dimerization protein 2 (JDP2) gene expression, cell proliferation and cell cycle were investigated by RT-qPCR and FACS analysis in shUHRF1 colon cancer cell line.

**Results:**

Downregulation of TIP60-mediated UHRF1 acetylation is correlated with suppressed cell cycle progression. Acetylation-deficient mimic of UHRF1 showed poor cell growth through increased expression of *JDP2* gene.

**Conclusions:**

Acetylation of UHRF1 4K residues by TIP60 is important for colon cancer cell growth. Furthermore, upregulated *JDP2* expression by acetylation-deficient mutant of UHRF1 might be an important epigenetic target for colon cancer cell proliferation.

## Introduction

Colon cancer is the third ranked leading cause of cancer deaths in the United States. Although various therapeutic strategies have been widely used to treat colon cancer, the 5-year survival rate of patients with all stages of colon cancer is only 22% (Siegel et al. 2022). Therefore, better understanding underlying of the mechanisms of colon cancer cell proliferation is needed to uncover novel therapeutic targets for colon cancer.

Ubiquitin-like with PHD and RING finger domains 1 (UHRF1) is a multi-domain protein which has been originally known for its role in maintenance of DNA methylation. UHRF1 recognizes and binds hemi-methylated DNA and recruits DNMT1 to DNA replication foci (Arita et al. 2008; Bostick et al. 2007). UHRF1/DNMT1 also involve in promoter hypermethylation of tumor suppression genes (TSGs) to downregulate its expression and inhibits cellular apoptosis (Alhosin et al. 2011). Furthermore, recent studies revealed that UHRF1 has a role in DNA damage repair process which depends on its posttranslational modifications (PTMs) (Hahm et al. 2019; Zhang et al. 2016).

Tat interactive protein, 60 kDa (TIP60) also known as lysine acetyltransferase 5 (KAT5), is one of the MYST family of histone acetyltransferases (HATs). TIP60 participates in a variety of cellular processes such as chromatin remodeling, DNA damage repair, gene transcription regulation, cell cycle and apoptosis (Gorrini et al. 2007; Idrissou et al. 2018; Ikura et al. 2000; Sapountzi et al. 2006). Moreover, TIP60 acetylates non-histone proteins like ATM and XPF-ERCC1 complex to regulate cellular responses such as DNA double-strand breaks (DSBs) or interstrand crosslinks (ICLs), respectively (Sun et al. 2005; Wang et al. 2020).

In this work, we identified that UHRF1 is acetylated by TIP60 at four lysine residues in SRA-RING domain (K644, K646, K648, K650) and acetylation-deficient UHRF1 mutant impeded colon cancer cell growth through re-expression of TSGs such as Jun dimerization protein 2 (JDP2), which acts as a transcriptional regulator for cell growth and cell cycle progression (Tsai et al. 2016).

## Materials and methods

### Cell culture

293T cells were grown in Dulbecco’s modified Eagle’s medium (DMEM, Gibco), and HCT116 cells were grown in Roswell Park Memorial Institute (RPMI) 1640 medium  (RPMI 1640, Gibco) containing 10% fetal bovine serum (FBS, Gibco) and 0.05% penicillin–streptomycin (Welgene) at 37 ℃ in 5% CO_2_. 293T and HCT116 cells were transfected with the corresponding DNA constructs using polyethyleneimine (PEI, Polyscience).

### Western blot analysis

Total lysates were prepared from cells using NP40 lysis buffer (50 mM Tris-HCL [pH 8.0], 150 mM NaCl, 0.1% SDS, 0.5% SDC, 1% NP40, 0.5 × protease inhibitor cocktail, 1 mM EDTA [pH 8.0]). The lysates were rotated 30 min at 4 ℃, sonicated on ice for 10 s and centrifuged. The lysates were fractionated on sodium dodecyl sulfate-polyacrylamide gel electrophoresis (SDS-PAGE) gel and transferred to nitrocellulose membranes. The membranes were probed at 4 ℃ overnight with the primary antibodies anti-UHRF1 (1:1000, sc-373750, Santa Cruz), anti-TIP60 (1:500, sc-166323, Santa Cruz), anti-GFP (1:1000, sc-9996, Santa Cruz), anti-mouse IgG (1:1000, sc-2025, Santa Cruz), anti-FLAG® M2 (1:10000, F3165, Sigma), anti-Rabbit IgG (1:5000, 12–370, Merck) and anti-β-actin (1:1000, sc-47778, Santa Cruz). The blots were incubated with horseradish peroxidase (HRP)-conjugated goat anti-rabbit or goat anti-mouse IgG (1:5000, Enzo Life Sciences) and detected using an ECL system (ABClon).

### *In vitro* acetylation assay

*In vitro* acetylation assays were performed at 30 ℃ for 3 h in 30 µL volumes containing 50mM Tris-HCl (pH 8.0), 50% Glycerol, 0.5 mM EDTA, 5 mM dithiothreitol, 100 nCi of [^14^C]-acetyl CoA (Perkin Elmer), GST-UHRF1 full-length (FL), UHRF1 deletion constructs GST-UHRF1 ∆1, ∆2, and ∆3, GST-UHRF1 4KR (K644R, K646R, K648R, K650R) mutant and 0.5 mg of GST-TIP60. Proteins were separated using SDS-PAGE and analyzed by autoradiography.

### Lentivirus transduction

To produce the lentiviral particles, 293T cells were co-transfected with plasmids encoding VSV-G and NL-BH, and the pLKO.1-TRC vector (Addgene) expressing shRNAs against TIP60 CDS (5′ – CCGGTCGAATTGTTTGGGCACTGATCTCGAGATCAG TGCCCAAACAATTCGATTTTTG − 3′), UHRF1 3′-UTR (5′ – CCGGAGATATAACGTT AGGGTTTCTCGAGAAACCCTAACGTTATATCTTTTTTG– 3′). Two days after the transfection, the supernatants containing the virus were collected and used to infect HCT116 cells in the presence of polybrene (8 µg/mL).

### Reverse transcription and real-time PCR

Total RNA was isolated from cells using Tri-RNA Reagent (Favorgen). After cDNA synthesis, the cDNA was quantified and subjected to mRNA expression analysis. Dissociation curves were examined after each PCR run to verify the amplification of a single product of the appropriate length. The mean threshold cycle (C_T_) and standard error values were calculated from individual C_T_ values obtained from triplicate reactions per stage. The normalized mean C_T_ value was estimated as ΔC_T_ by subtracting the mean C_T_ of β-actin. ΔΔC_T_ values were calculated as the difference between the control ΔC_T_ and the values obtained for each sample. The n-fold changes in gene expression relative to an untreated control were calculated as 2^−ΔΔCT^. The following primers were used in this study : *JDP2* Forward 5′-CTG TGG AGG AGC TGA AAT AC-3′ Reverse 5′-TCC TCT TCC TCA TCT AGC TC-3′, *FBLN2* Forward 5′-TGG CGT GTC CTG TGA AGA CAT-3′ Reverse 5′-GAG TGC CTT GTA GCA GTG GAA-3′, *UCHL1* Forward 5′-ATG TCG GGT AGA TGA CAA GGT G -3′ Reverse 5′- AGC AGG GTG TCC TCT GAA CT -3′.

### MTT assay

Control and shUHRF1 HCT116 cells were seeded at a density of 5 × 10^3^ cells/well in 48-well plates. After 24, 48, 72, and 96 h, MTT was added to the cells (200 µL, final concentration 0.5 mg/mL), and the cells were incubated for a further 2 h at 37 °C. The medium was then removed, and DMSO was added (200 µL). The absorbance at 575 nm was determined using a spectrophotometer.

### Colony formation

UHRF1 stable knockdown and control cells were seeded at a density of 2 × 10^3^ cells/well in a six-well culture plate, transfected with GFP-UHRF1 WT and 4KR, and incubated for 7 days. Surviving colonies were fixed with methanol, stained with crystal violet, and counted.

### Immunoprecipitation assay

Cells were lysed in lysis buffer (50 mM Tris-HCl (pH 7.5), 200 mM NaCl, 0.5% NP-40, 1 × protease inhibitor cocktail) and incubated with indicated antibodies overnight at 4 °C. Protein A/G agarose beads (GenDEPOT) were then added, and the mixture was mixed for 3 h at 4 °C. Bound proteins were then analyzed by immunoblotting.

### Statistical analysis

The data were expressed as the mean ± standard error of the mean (SEM) of three independent experiments. Data were analyzed using GraphPad Prism (version 7; GraphPad Software). Statistically significant effects (*P* < 0.05) were evaluated using Microsoft EXCEL software. Differences between groups were evaluated via Student’s *t*-tests.

## Results

### UHRF1 interacts with TIP60

**Fig. 1:**
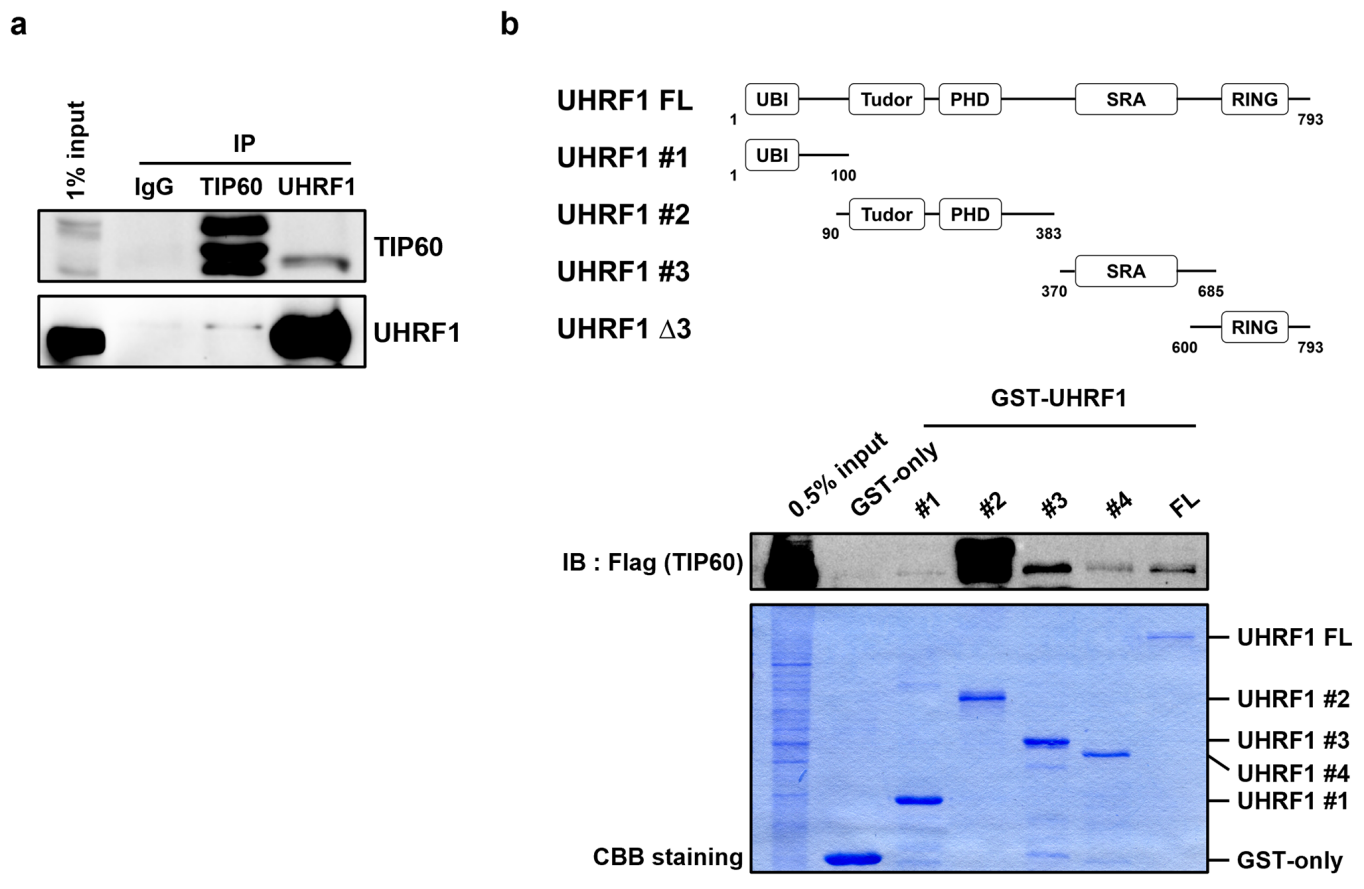
UHRF1 interacts with TIP60. **a** HCT116 cells were immuno-precipitated with control immunoglobulin G (IgG), anti-UHRF1, or anti-TIP60 antibodies and evaluated by western blots. **b** Schematic representations of UHRF1 and its functional domains (top). FLAG-TIP60 was transfected into HCT116 cells. The cell extracts were incubated with purified each GST-fusion deletion mutants of UHRF1 (bottom).

Previously, we identified that UHRF1 is acetylated by PCAF and inhibits its binding to hemi-methylated DNA in HCT116 cell line (Hahm et al. 2020). Another study revealed that TIP60 acetylated UHRF1 at K659 (isoform 1, K644 at isoform 3) by *in vitro* acetylation assay (Zhang et al. 2015). However, there has been no report whether UHRF1 could be acetylated by TIP60 *in vivo*. Since both UHRF1 and TIP60 are localized in the nucleus and involved in DNA damage repair and cell proliferation (Squatrito et al. 2006; Zhang et al. 2016), we first investigated the interaction between UHRF1 and TIP60 *in vivo*. To determine whether UHRF1 interacts with acetyltransferase TIP60, immunoprecipitation (IP) assay was performed in HCT116 cells. We could identify that TIP60 interacts with UHRF1 *in vivo* (Fig. 1a). Then, GST-pull down assays were performed using GST tagged UHRF1 FL and partial recombinant proteins to find which domain interacts with TIP60. UHRF1 Tudor-PHD, SRA, RING domain, which bearing 90–793 amino acids, is interacted with TIP60. UHRF1 Tudor-PHD domain showed very strong interaction with TIP60, and SRA and RING domain also showed interactions with TIP60 (Fig. 1b). Together, these data demonstrated that UHRF1 interacts with TIP60 both *in vitro* and *in vivo*.

### UHRF1 is acetylated by TIP60

**Fig. 2:**
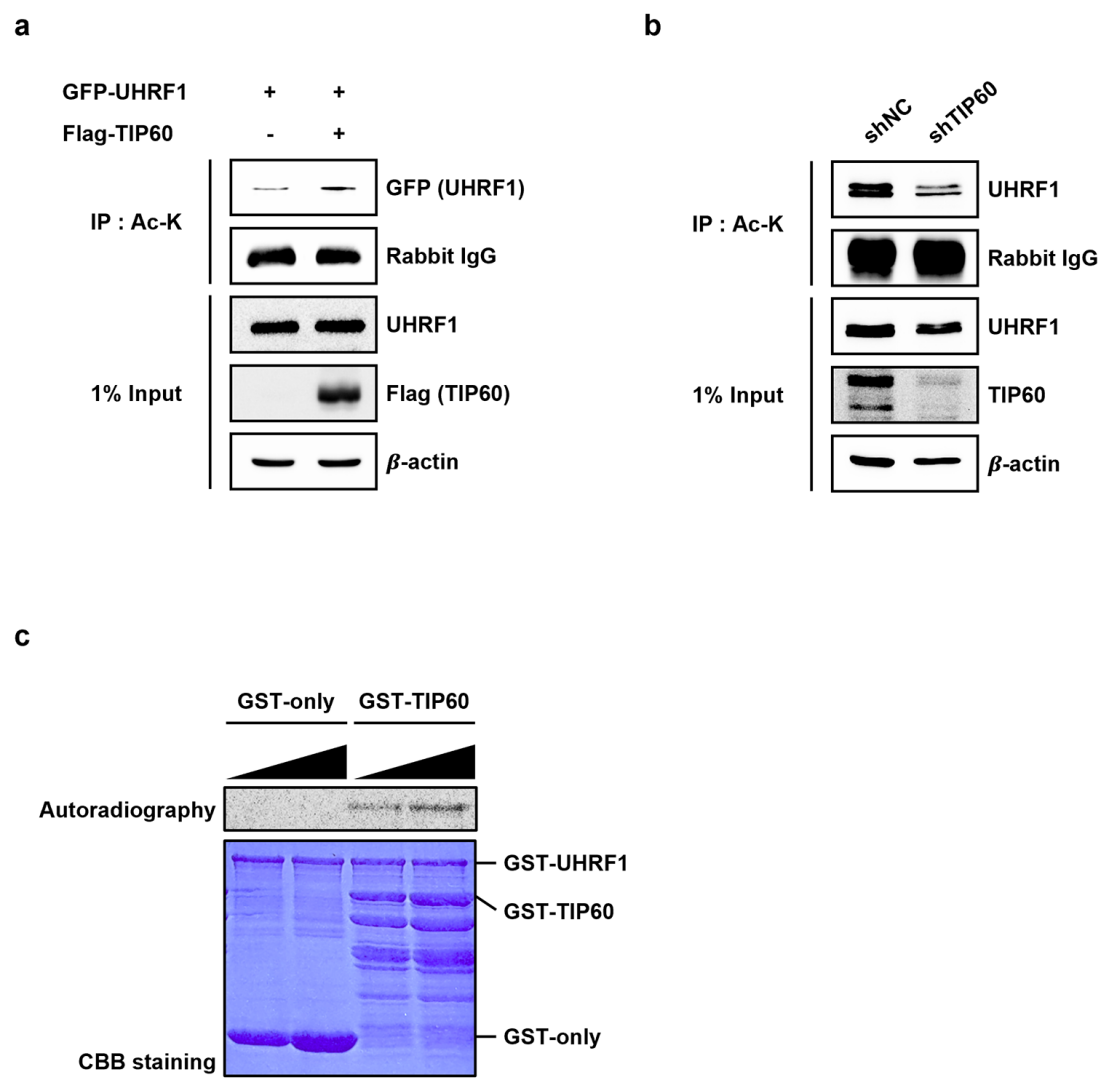
UHRF1 is acetylated by TIP60. **a** GFP-UHRF1 and FLAG-TIP60 were co-transfected into HCT116 cells. The cell lysates were immunoprecipitated with anti-pan-acetyl antibodies and immunoblotted with indicated antibodies. **b** TIP60 was stably knocked down in HCT116 cells. HCT116 cell extract of control and shTIP60 were immunoprecipitated with anti-pan-acetyl antibodies and immunoblotted with indicated antibodies. **c** Full-length GST-fusion UHRF1 was incubated with [^14^C]-acetyl-CoA and GST-fusion TIP60 for 3 h at 30 ℃. The samples were separated by SDS-PAGE and stained with Coomassie brilliant blue (CBB) or visualized by autoradiography.

Having identified interaction between UHRF1 and TIP60, we next investigated whether UHRF1 is acetylated by TIP60 *in vivo*. Overexpression of FLAG-TIP60 increased acetylation of GFP-UHRF1 (Fig. 2a). Consistently, stable knockdown of TIP60 using shRNA showed decreased acetylation of UHRF1 by IP assay using pan-acetylation antibody (Fig. 2b). Then, *in vitro* acetylation assay was performed using full-length GST tagged TIP60 and UHRF1 protein. The assay result shows that GST-UHRF1 was acetylated by TIP60 in a dose-dependent manner (Fig. 2c). These data suggests that UHRF1 is acetylated by TIP60 both *in vitro* and *in vivo*.

### TIP60 acetylates UHRF1 4 K residues in SRA-RING domain

**Fig. 3:**
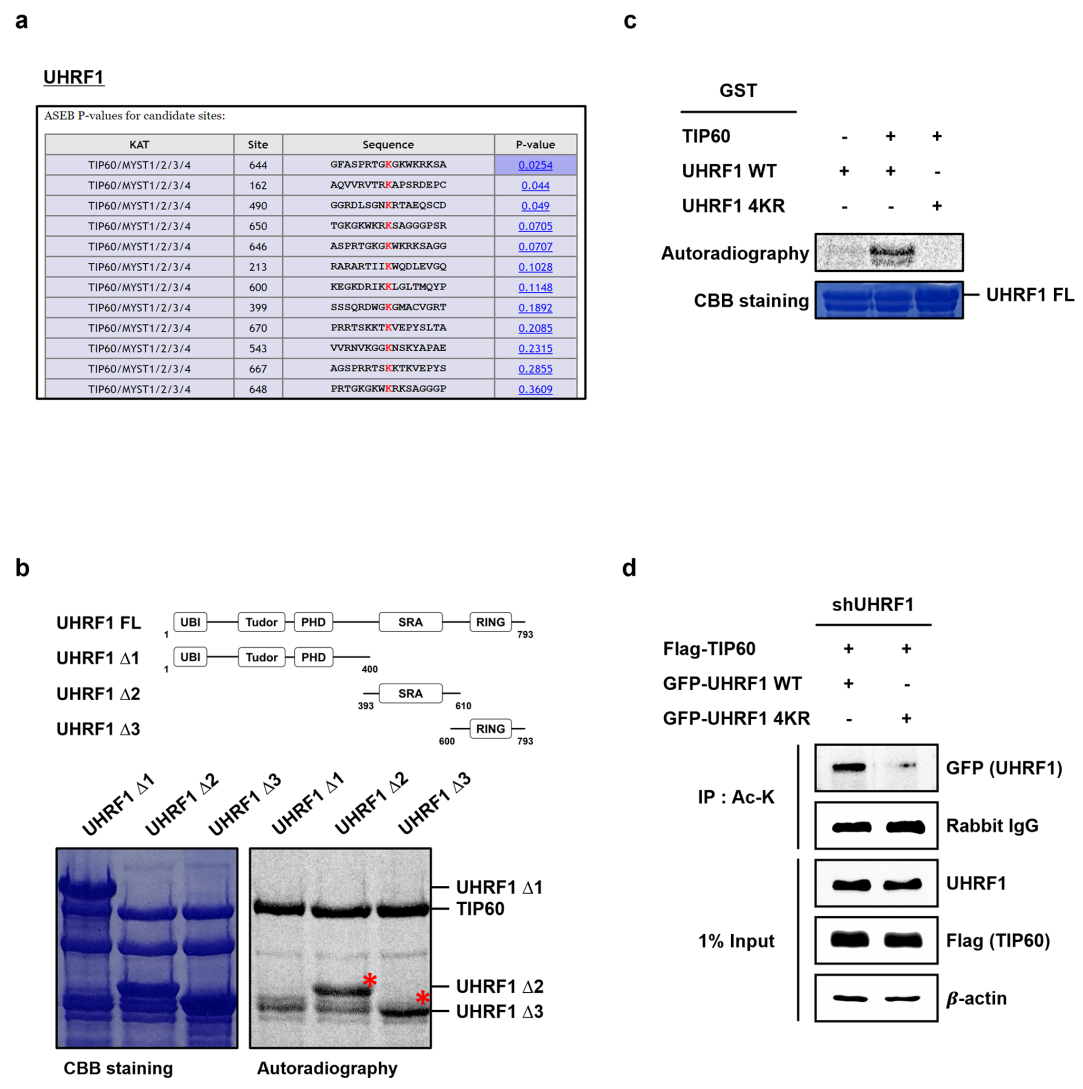
TIP60 acetylates UHRF1 4KR residues of SRA-RING domain. **a** TIP60-mediated acetylated lysine residues within UHRF1 protein were predicted by a web server for KAT-specific Acetylation Site Prediction (ASEB). **b** Partial recombinant constructs of UHRF1 were used as substrates for *in vitro* acetylation assays with GST-TIP60. * means acetylated UHRF1. **c** WT and 4KR of UHRF1 protein were incubated with and [^14^C]- acetyl-CoA and recombinant TIP60 for 3 h at 30 ℃. The samples were separated by SDS-PAGE and stained by CBB or exposed by autoradiography. **d** HCT116 shUHRF1 cells overexpressing TIP60 and UHRF1 WT or UHRF1 4KR were immunoprecipitated using anti-pan-acetyl antibodies and immunoblotted with indicated antibodies.

To predict TIP60-mediated UHRF1 acetylation sites more precisely, we used a webserver for KAT-specific Acetylation Site Prediction for ASEB (Acetylation Set Enrichment Based) method (Wang et al. 2012b). Possible UHRF1 acetylation sites by TIP60 were presumed by this method. Of these, UHRF1 K644 residues inside SRA-RING domain could be the most probable acetylation sites by TIP60 (Fig. 3a). Subsequently, we performed *in vitro* acetylation assay using full-length and different partial construct of GST-UHRF1 and found that TIP60 acetylates SRA domain (UHRF1 ∆2) and RING domain (UHRF1 ∆3) of UHRF1 (Fig. 3b). Since the 644–650 amino acid sequence of UHRF1 was lysine-rich region, we focused on these predicted residues and generated UHRF1 quadruple mutant in which 4 lysine residues (K644, K646, K648, K650) were replaced with arginine (UHRF1 4KR). *In vitro* acetylation assay using UHRF1 4KR mutant showed significantly reduced acetylation level compared to that of UHRF1 wild type (WT) (Fig. 3c). Furthermore, IP assay with co-transfection of TIP60 and UHRF1 WT or UHRF1 4KR into UHRF1 stable knockdown cells exhibited dramatic decrease in acetylation level in UHRF1 4KR-expressing cells (Fig. 3d). These results suggest that UHRF1 4 K (K644, K646, K648, K650) residues analyzed are primary candidates for acetylation sites by TIP60.

### TIP60-mediated UHRF1 acetylation is crucial for colon cancer cell proliferation

**Fig. 4:**
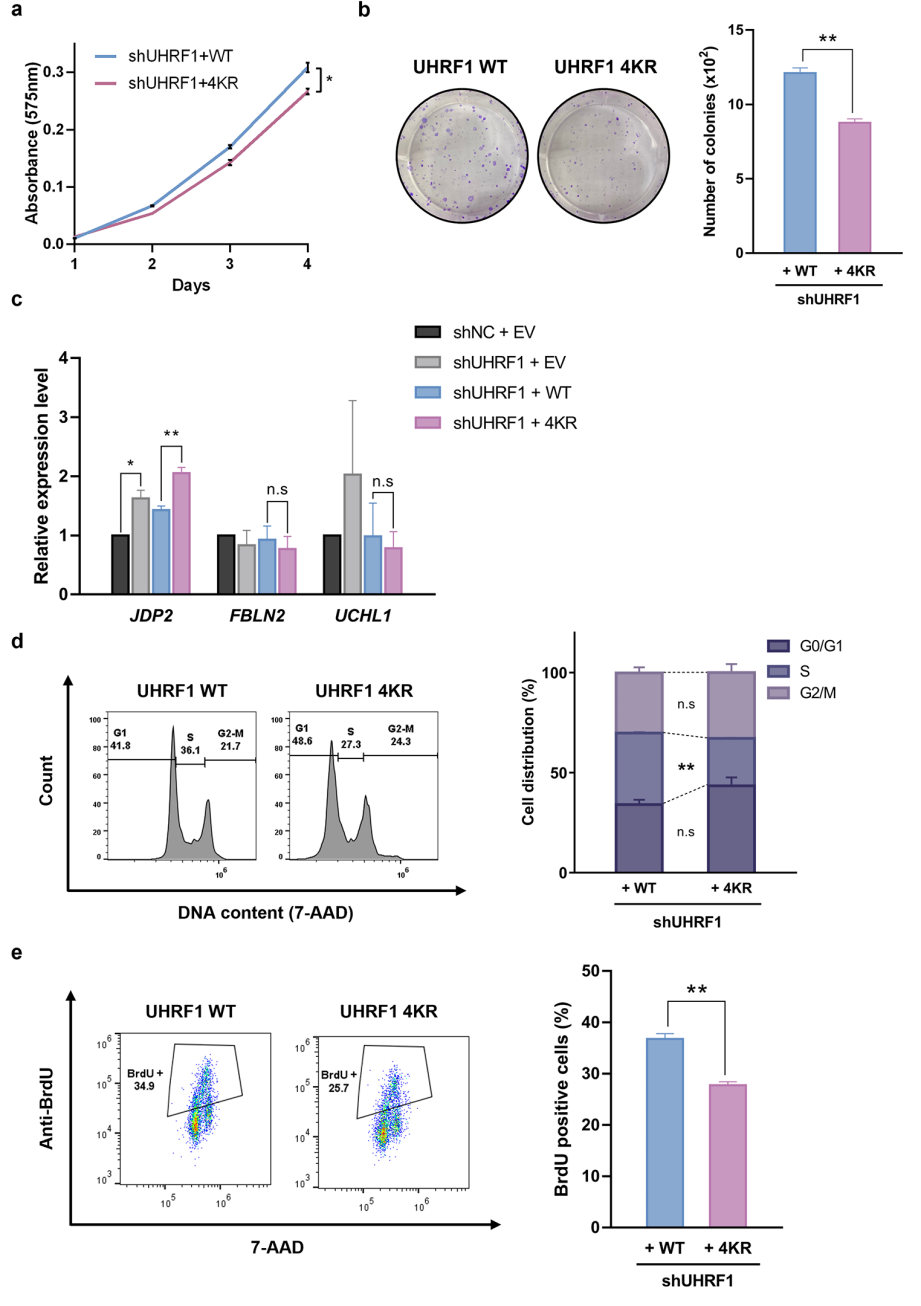
TIP60-mediated UHRF1 acetylation is crucial for cell proliferation. **a** Cell viability was determined by MTT assay. HCT116 shUHRF1 cells were transfected with UHRF1 WT or UHRF1 4KR. Results were shown as mean ± SEM, *n* = 3; *p < 0.05. **b** Representative colony formation assay using HCT116 shUHRF1 cells expressing UHRF1 WT and UHRF1 4KR. Cells were incubated in fresh media for 7 days. Results were shown as mean ± SEM, *n* = 3; **p < 0.01. **c** RT-qPCR analysis of *JDP2, FBLN2, UCHL1* gene using HCT116 control and shUHRF1 cells transfected with EV, UHRF1 WT and 4KR. Results were shown as mean ± SEM, *n* = 3; *p < 0.05, **p < 0.01, n.s: no significant difference. **d** HCT116 shUHRF1 cells transfected with UHRF1 WT and 4KR were fixed and stained with 7-Aminoactinomycin D (7-AAD), and the DNA content was measured by fluorescence-activated cell sorting (FACS) analysis. Data are presented as mean ± SEM, *n* = 3; **p < 0.01, n.s: no significant difference. **e** HCT116 shUHRF1 cells transfected with UHRF1 WT and 4KR were treated with 10 µM BrdU using pulse labelling for 30 min were fixed, immunostained with anti-BrdU-APC for 1 h and stained with 7-AAD for 5 m. BrdU-positive cells were measured by FACS analysis. Data are presented as mean ± SEM, *n* = 3; **p < 0.01.

In the previous study, we showed that UHRF1 K490 acetylation through PCAF impairs hemi-methylated DNA binding of UHRF1 (Hahm et al. 2020). To identify whether TIP60-mediated acetylation of UHRF1 could induce any physiological effect on HCT116 cells, we performed MTT assay and colony formation assay with shUHRF1 by comparing transfection of UHRF1 WT and 4KR mutant. Surprisingly, UHRF1 knockdown cells recovered with UHRF1 acetylation-deficient mutant (UHRF1 4KR) had inhibited cell proliferation compared to those recovered with UHRF1 WT (Fig. 4a, b), suggesting the important role of TIP60-mediated acetylation of UHRF1 in HCT116 cell proliferation.

Recent study indicates that UHRF1 acts as an oncogene by silencing 8 selected TSGs in colon cancer cells through promoter hypermethylation of these TSGs and correlated with poor prognosis and the shortened overall survival rates (Kong et al. 2019). Since we found UHRF1 4KR effect on colon cancer cell proliferation, we tested whether UHRF1 acetylation-deficient form could alter expression level of these TSGs by RT-qPCR. We selected 3 out of 8 genes which could function as TSGs through inhibition of cell cycle or cell proliferation in cancer cells. Among these 3 TSGs, UHRF1 4KR failed to suppress *JDP2* expression compared to that of UHRF1 WT (Fig. 4c). And we confirmed these changes in *JDP2 *expression are TIP60-dependent through qRT-PCR by using stable KD shTIP60 cells (Data not shown). Given that knockout of *JDP2* is correlated with increased S phase cells though upregulation of cyclin-A2 (*ccna2*) mRNA level which is essential for cell cycle progression (Pan et al. 2010), we performed cell cycle analysis of UHRF1 WT and 4KR mutant by FACS. Cell cycle analysis indicates that UHRF1 4KR exhibited reduced distribution of S-phase cells compare to UHRF1 WT (Fig. 4d). Correspondingly, BrdU assay was performed by FACS to measure replicating DNA. UHRF1 4KR mutant exhibited reduced incorporation of BrdU, which indicates the arrest of HCT116 cell proliferation (Fig. 4e). These findings suggests that the acetylation-deficient form of UHRF1 impedes DNA replication through re-expression *JDP2*, therefore it is contributed to inhibition of HCT116 cell growth. Overall, these results suggests that UHRF1 acetylation by TIP60 is very important for the proliferation of HCT116 cells.

## Discussion

Studies suggest that UHRF1 is upregulated in different cancer cells (Unoki et al. 2009; Wan et al. 2016), another study determined the oncogenic propriety of UHRF1 on colon cancer cell proliferation (Kofunato et al. 2012). Cellular function of UHRF1 is controlled by several posttranslational modification (PTMs) such as methylation, phosphorylation, ubiquitination, and acetylation and they can impact cell cycle progression and cell proliferation regulation (Ma et al. 2012; Zhang et al. 2019).

In this study, we discovered that UHRF1 interact with TIP60. And IP and *in vitro* acetylation assays revealed that UHRF1 is directly acetylated at 4K (K644, K646, K648, K650) by TIP60, which is in the lysine-rich region of SRA-RING domain.

Several studies sought to reveal the function of UHRF1 on TSGs in multiple cancer cells such as lung and gastric cancer and these TSGs were silenced though its promoter hypermethylation (Daskalos et al. 2011; Zhou et al. 2015). Representatively, the expression of tumor suppresser gene *p16*^INK4A^ ,which is involved in the G1/S cell cycle checkpoint, is being silenced by UHRF1-mediated epigenetic modifications like promoter hypermethylation. Knockdown of UHRF1 contributed to re-expression of *p16*^INK4A^ (Wang et al. 2012a), indicating oncogenic function of UHRF1 could be linked to regulate expression of TSGs. However, whether the role of UHRF1 in suppressing TSGs can be regulated by its PTMs is not fully identified.

Here, using UHRF1-deficient HCT116 cells transfected with UHRF1 WT or acetylation-deficient mutant 4KR, we found that UHRF1 4KR failed to suppress tumor suppressor gene *JDP2* as much as shUHRF1. *JDP2* gene is known as one of UHRF1 target genes that correlates with increased UHRF1 expression and promoter DNA methylation in colon cancer cells (Kong et al. 2019) and also known for inhibiting cancer cell proliferation by repressing cell cycle progression (Heinrich et al. 2004). We analyzed cell cycle between UHRF1 WT and 4KR and identified that 4KR affects colon cancer cell growth through re-expression of *JDP2*. Although we suggest that acetylation of UHRF1 can regulate expression of tumor suppressor *JDP2* gene, further studies are needed to determine whether UHRF1 4KR mutant can inhibit *JDP2* expression through promoter hypermethylation or other epigenetic modifications.

Studies to find other TSGs regulated by UHRF1 in colon cancer have been continued (Taniue et al. 2020), our findings could contribute to reveal the oncogenic function of UHRF1.
